# Shedding Light on Capillary-Based Backscattering Interferometry

**DOI:** 10.3390/s22062157

**Published:** 2022-03-10

**Authors:** Niall M. C. Mulkerns, William H. Hoffmann, Ian D. Lindsay, Henkjan Gersen

**Affiliations:** 1 H. H. Wills Physics Laboratory, University of Bristol, Bristol BS8 1TL, UK; niall.mulkerns@bristol.ac.uk (N.M.C.M.); w.hoffmann@bristol.ac.uk (W.H.H.); i.d.lindsay@bristol.ac.uk (I.D.L.); 2 Bristol Centre for Functional Nanomaterials, University of Bristol, Bristol BS8 1TL, UK; 3 School of Chemistry, University of Bristol, Bristol BS8 1TS, UK

**Keywords:** backscattering interferometry, refractive index, capillary, ray tracing

## Abstract

Capillary-based backscattering interferometry has been used extensively as a tool to measure molecular binding via interferometric refractive index sensing. Previous studies have analysed the fringe patterns created in the backscatter direction. However, polarisation effects, spatial chirps in the fringe pattern and the practical impact of various approximations, and assumptions in existing models are yet to be fully explored. Here, two independent ray tracing approaches are applied, analysed, contrasted, compared to experimental data, and improved upon by introducing explicit polarisation dependence. In doing so, the significance of the inner diameter, outer diameter, and material of the capillary to the resulting fringe pattern and subsequent analysis are elucidated for the first time. The inner diameter is shown to dictate the fringe pattern seen, and therefore, the effectiveness of any dechirping algorithm, demonstrating that current dechirping methods are only valid for a subset of capillary dimensions. Potential improvements are suggested in order to guide further research, increase sensitivity, and promote wider applicability.

## 1. Introduction

Backscattering interferometry (BSI) has been widely adopted as a tool to measure the binding kinetics of receptor–guest systems in many different modalities [1,2,3,4]. In addition to this, BSI has also found extensive use as a refractive index sensor [5,6,7,8] with limits of detection in some modalities down to $10^{-9}$ refractive index units (RIU) [3]. The simplicity of BSI coupled with the ability to measure free-solution binding makes the technique attractive in many analytical scenarios [9]. Whilst finding great success in this research field, BSI does not have a unified model that is independent of commercial software [10,11]. To further improve the technology, every aspect of BSI must be analysed to leverage all the information available. For example, the effect of the polarisation state of the incident light on the final fringe pattern for all capillary dimensions has never been fully described [11]. In addition, the literature on ray tracing simulations is conflicting in certain situations and would benefit from clarification.

Here, BSI comprising a hollow glass tube is addressed and the fringe pattern that is created is analysed, with the goals of gaining a better understanding of and improving further the sensitivity of the technique. This geometry has been used extensively up to the present day [12], though semicircular microfluidic channels have been adopted more recently [13]. This new system is significantly easier to analyse theoretically due to its similarity to other interferometric devices, such as Fabry-Pérot etalons and Michelson interferometers at normal incidence. The downsides to the microfluidic approach are the higher manufacturing costs and the minimal increase in sensitivity and stability in its current implementation [3,4].

In this paper, two ray tracing models [7,14] are combined to create a composite model, and are analysed in reference to experimental data. These two models have multiple differences in their construction, which will be explored and analysed in detail. Modifications to the composite model to facilitate the inclusion of explicit polarisation dependence are implemented, comparing the simulations to experimental data. Finally, perspectives on how a deeper theoretical understanding can improve the data and analysis of BSI are given.

## 2. Materials and Methods

### 2.1. Experimental Apparatus

**Figure 1:**
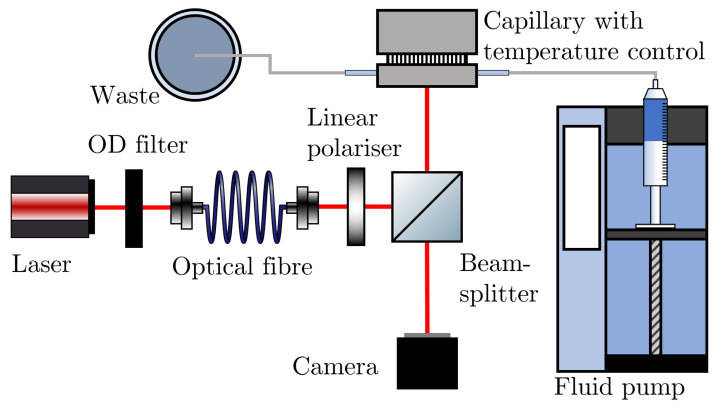
A schematic diagram of the backscattering interferometry apparatus used in this work.

Backscattering interferometry is an optical technique where the interference of reflected and refracted light from a glass capillary is utilised to remotely sense the refractive index of a solution held within the capillary. The BSI apparatus used in this paper is shown in Figure 1 and was custom built based on the system set out by Sørensen et al. [15], albeit with minor differences.

A laser (JDS Uniphase 1137P, 7 mW) is passed through an optical fibre (ThorLabs, S405-XP) and linear polariser (ThorLabs, LPVISE100-A) to obtain an single spatial mode with a linear polarisation state. This light is incident on a circular cross-section glass capillary (Drummond, ID 0.556 mm, OD 0.800 mm, and ID 0.343 mm, OD 0.864 mm) of some outer radius *R* and inner radius *r* with refractive index $n_{1}$. The custom aluminium stage is held at a constant temperature by a Peltier thermoelectic module (Laird annular SH-10 controlled by Wavelength Electronics WTC3243HB) and holds the capillary which contains a liquid of refractive index $n_{2}$ that can be exchanged using a fluid pump (Cole-Parmer Masterflex). The light that is reflected and refracted from the capillary in a direction antiparallel to the incident light forms an interference pattern collected by a camera (Blackfly BFS-U3-200S6C-C) at some horizontal and vertical distance to the capillary. This interference pattern is recorded using a custom LabVIEW script as the fringes move laterally due to changes in the refractive index of the liquid inside the capillary. In this work, the fringe translation is tracked by monitoring the phase change of the desired frequency peak in a Fourier transform of the intensity pattern; however, other methods, such as bi-cells [3], have been previously employed.

### 2.2. Model Overviews

Ray tracing is a optical simulation technique based on propagating a given ray of light through the system in question. The application of simple geometrical and optical transformations at interfaces allows parameters such as amplitude, optical phase, and propagation direction to be determined analytically at any point along the path. Ray tracing is a common technique that has been extensively applied to the simulation of light in computer-generated imagery [16], and the optical design of novel materials [17]. As this method assumes light to be a ray, phenomena that rely on wavefront effects (e.g., diffraction) will not be accounted for. This limitation should not invalidate the simulations of BSI presented here due to the macroscopic size of all components [18,19]. Other papers have undertaken more extensive ray tracing simulations [10,11] using commercial software; however, few details of these models are given, making a comparison of methods virtually impossible.

The detailed mathematics of both the Tarigan and Xu/You methods can be found in their respective papers [7,14,20]; however, due to the similarities of the methods and to aid discussion, a simplified overall explanation is given here. Additionally, a full overview of the unified model used in this paper is given in the Supplementary Materials.

### 2.3. Ray Choices

**Figure 2:**
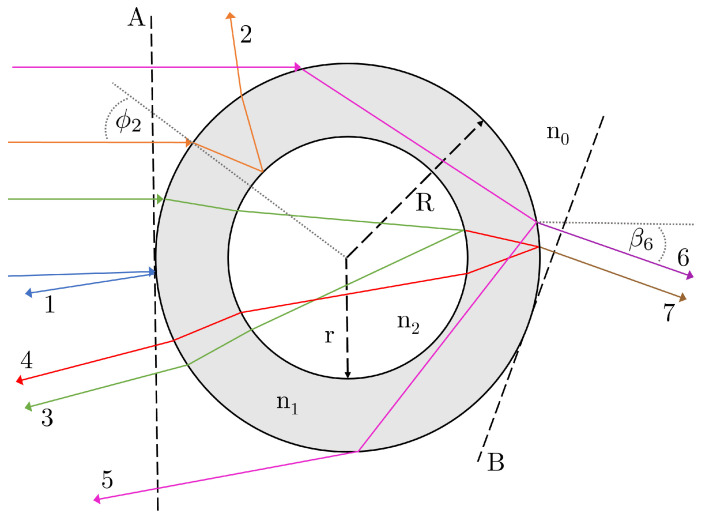
A diagram showing the different first-order rays that are possible in the capillary geometry of BSI. The incident angle of a given ray $\phi_{i}$ is simply the angle to the surface normal. In the Xu/You et al.’s methodology, the angle of incidence is related to the viewing angle $\beta_{i}$ of each ray, where $\beta_{i}$ is defined to be the angle to the horizontal in this geometry. The example of $\beta_{6}$ is shown here. The diagram is adapted from that in You et al. [20]. The rays are demonstrative and not drawn with geometric accuracy.

A depiction of the cross-sectional geometry of the system is given in Figure 2. Light impinging on a capillary of inner radius *r* and outer radius *R* is assumed to be in the form of a plane wave of equal intensity at all incident angles. As the capillary has a circular external cross-section, the incident angle of a given ray $\phi_{i}$ is linked to the vertical position of the ray, and can be considered the independent variable. It should be noted that the system here does not include the thin polyimide coating present on many capillaries used in the literature [14,15,21], so care should be taken when drawing comparisons.

As shown in Figure 2, there are potentially seven different first-order rays (i.e., those with non-negligible intensities) that may or may not contribute to the signal seen in the BSI. Each ray, denoted by its numeral *i*, can be converted from an input angle $\phi_{i}$ to an output or viewing angle $\beta_{i}$, depending on the path that it takes through the capillary. At some angles of incidence, the existence of a given ray number will no longer be physically possible (for example, at small angles of incidence $\phi_{i}$, ray 5 will be blocked by the inner wall and convert to a ray of number 1, 2, 3, 4, or 7); therefore, each ray will have bounds for which it is defined. This gives rise to the first discrepancy between the two methodologies—the number and description of the rays that are considered relevant. Tarigan et al. [14] use a four beam model, rays 1, 2, 3, and 4 in Figure 2, whereas Xu et al. [7] and You et al. [20] consider the more general problem of all 7 beams, shown in Figure 2. Most notably, then, the difference lies in the omission of ray 5 without explicit justification, as ray 6 and 7 do not contribute to the signal seen at β=π (antiparallel to the incident light, or “backscattered”). As previously mentioned, models with >5 rays for BSI do exist, but they are confined to commercial software [10,11].

Taking beam 5 as an example, the repeated usage of Snell’s law and geometrical arguments will yield the viewing angle $\beta_{5}$ in terms of $\phi_{5}$ and known constants:

(1) $$\beta_{5}=2\phi_{5}-4\arcsin\left(\frac{\sin\phi_{5}}{n_{1}}\right)+\pi ,$$

which is valid in the regime where $\arcsin\left(n_{1}r/R\right)\;\leq \;\phi_{5}\;\leq \pi /2\;$. At $\rho \;=\;n_{1}r/R\;>\;1$, ray 5 will cease to exist due to the ray being blocked by the inner diameter. For a typical fused silica capillary, the refractive index will be $n_{1}\;\approx \;1.457$ at 632.8 nm, meaning that r/R < 0.68 for ray 5 to be valid. This vanishing of ray 5 at high inner-to-outer radius ratios is not alluded to in any implementations of BSI, despite the r/R ratio of many previously used experimental systems being very close to the r/R = 0.68 limit [22] or below it [10]. Having said that, both Jørgensen et al. [18] and Tarigan et al. [14] use capillaries above this limit, and so, correctly, do not consider ray 5 in their work. Note that, in most cases, the refractive index of the capillary is unknown, and so r/R = 0.68 is simply an estimate. It should also be noted that the disappearance of ray 5 is not instantaneous; as r/R→0.68, ray 5 will be defined for a smaller angular range centered on $\beta_{5}\;=\;\pi$, until at r/R = 0.68, it will no longer be defined for any angle.

The small angle approximation can simplify Equation (1) and the other angular transformations, which are leveraged by Tarigan et al. [14]. This simplification is justified when considering only ray 1, 2, 3, and 4; however, if r/R < 0.68, the small angle approximation cannot be assumed for ray 5 as, in this case, only large angles contribute to the final answer (see Figure 2). In addition, the simplification by Tarigan et al. that the path length for rays 1–4 for the portion in the glass is $\left(R\;-\;r\right)n_{1}$ causes discrepancies to be introduced between the two methods at incident angles above ∼10°. By changing this factor to the geometrically accurate version, as is done in this paper (see Supplementary Materials), this discrepancy is completely eliminated. Lastly, comparing the two methods highlights that a factor of two is missing from the Tarigan method in all the angle transformation formulae. This alters the frequency of each interference component, although this is a systematic error that does not impact the underlying physics or computed sensitivities.

An additional complexity arises from the fact that a central assumption in the Xu et al. and You et al. derivations is that the refractive index of the liquid within the capillary is higher than that of the capillary itself, $n_{2}\;>\;n_{1}$. This assumption is invalid for typical BSI experiments, as the solvent used for biological systems is primarily water ($n_{2}∼1.3\;-\;1.4$) and the capillary material is glass ($n_{1}∼1.5$). To correct this assumption, the angular bounds of rays 3, 4, and 7 must be adjusted [20] (see Supplementary Materials).

### 2.4. Interference

After the conversions between incident angle $\phi_{i}$ and viewing angle $\beta_{i}$, the relative intensities of the rays must be elucidated, and then overlapping beams interfered pairwise. To do this, the fractions of reflected and transmitted light must be calculated at each interface. In addition, for interference to occur, it must be ensured that the coherence length of the laser is greater than the largest possible path length difference between rays. As BSI typically employs helium–neon lasers with coherence lengths on the order of 10 cm, this criterion is satisfied for all ray pairs. Taking ray 5 as an example again, the ray undergoes a transmission from $n_{0}$ to $n_{1}$, a reflection from the interface of $n_{1}$ to $n_{0}$ at $\theta_{5}\;=\;\arcsin\left(\sin\left(\phi_{5}\right)/n_{1}\right)$, and then a transmission from $n_{1}$ to $n_{0}$ at the same angle $\theta_{5}$. Algebraically, this “scattering factor” $S_{i}$ for ray i = 5 can be written as:

(2) $$S_{5}=T_{r}\left(\phi_{5},n_{0},n_{1}\right)\;\cdot \;R_{e}\left(\theta_{5},n_{1},n_{0}\right)\;\cdot \;T_{r}\left(\theta_{5},n_{1},n_{0}\right),$$

where *T* and *R* denote power transmission and reflection as a function of incident angle and the refractive indices of the incident and boundary material, respectively.

**Figure 3:**
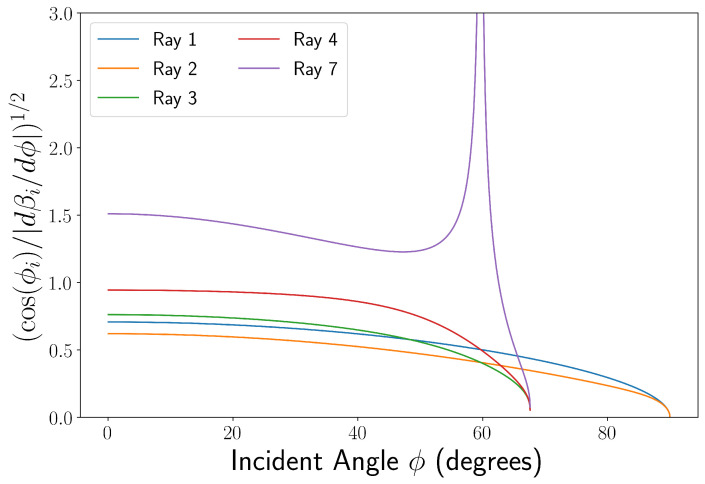
A graph showing the change of $f_{i}$ with incident angle as set out in Equation (3). All rays excluding ray 5 and ray 6 are shown here due to suppression from the capillary geometry considered. Parameters used were r/R = 0.74, $n_{1}\;=\;1.457$, $n_{2}\;=\;1.333$. The cut off for rays 3, 4, and 7 at $\phi_{i}∼70{}^{\circ}$ is due to these rays being unable to enter the core above this angle.

As stated by You et al. [7,20], an additional multiplicative factor $f_{i}$ for each beam equal to:

(3) $$f_{i}={\left(\frac{\cos(\phi_{i})}{|d\beta_{i}/d\phi |}\right)}^{1/2},$$

is included with the scattering factors to conserve the flux of the radiant energy due to the divergence of the light rays [19], where *i* indicates the ray number. The inclusion of $f_{i}$ is physically intuitive, as the viewing angles $\beta_{i}$ for each discrete input angle are approximately evenly spaced in the limit of low $\phi_{i}$, as the small angle approximation dictates. At larger incident angles, this is not true, however. For example, the light reflected from the exterior of the capillary (ray 1) will become infinitely spaced out as $\phi_{1}\to \pi /2$ [18]. However, as the inclusion of this term is rooted in the conservation energy density and Equation (2) is defined in terms of power [19], $S_{i}$ should instead be proportional to $f_{i}^{2}$. This $f_{i}$ factor is omitted in the derivation given by Tarigan [14] and Xu et al. [7]. Despite this, as shown in Figure 3, each $f_{i}$ is slowly varying and has a similar magnitude at small angles of incidence for rays 1–4, meaning that this factor is generally a simple scaling factor for these models. If directly comparing magnitudes of fringe patterns or when considering larger incident angles, the omission of $f_{i}$ may cause errors. The factor in Equation (3) is applied to the amplitude formulations of $S_{i}$ (see Supplementary Materials) for the simulations shown in this paper and can be derived from the equations for $\beta_{i}$ given in the Supplementary Materials.

The pathlengths of rays 1–7 are analytically determined from the plane normal to both the incident ray and the capillary exterior (dashed line A in Figure 2), to the plane normal to both the exit angle of the ray chosen and the capillary exterior (dashed line B in Figure 2 for the case of ray 6). Once again, taking the example of ray 5, the optical path length $L_{5}$ can be expressed as:

(4) $$L_{5}=2R\left[1-\cos(\phi_{5})\right]+4n_{1}R\cos\left(\theta_{5}\right),$$

where $\theta_{5}\;=\;\arcsin\left(\sin\left(\phi_{5}\right)/n_{1}\right)$.

To determine the fringe pattern seen when projected onto a camera, the beams must be interfered with each other pairwise. The total intensity *I* seen at some viewing angle β is given by:

(5) $$I\left(\beta \right)=\sum_{i=1}^{7}\sum_{j=i}^{7}\sqrt{I_{i}I_{j}}\cos\left(k\left[L_{i}-L_{j}\right]\right),$$

where $I_{i}\;=\;I_{0}S_{i}f_{i}^{2}$, with $I_{0}$ defined as the initial beam intensity (assumed constant with $\phi_{i}$ and beam number, here set to be $I_{0}\;=\;1$ for simplicity), and k = 2π/λ, with λ as the wavelength of incident light in vacuum. In Equation (5), the values of $L_{i}$ and $I_{i}$ are evaluated at values of $\phi_{i}$ that give rise to the exact same viewing angle β.

It is pertinent to note, at this stage, that this formulation of scattering factors $S_{i}$ typically neglects the phase changes introduced during reflection that are normally accounted for by using the amplitude versions of the reflection and transmission coefficients. Tarigan et al. explicitly accounts for this by adding in λ/2 shifts to the path lengths upon reflection; however, it is unclear to us whether this is factored into the analysis of Xu et al. In addition, the reflection and transmission are angle- and polarisation-dependent. Tarigan et al. assumes that the reflectance is normal at every incident angle used in BSI simulation. This assumption is valid where only small angles are considered. Normal reflection is polarisation-independent, though adoption for all angles will introduce a negligible intensity difference for all beams (up to 0.15% by 10°). You et al. use angular-dependent reflectance in power; however, they implicitly assume *s*-polarisation and do not consider *p*-polarisation. Here, the amplitude versions of the reflection and transmission coefficients are used, with phase changes upon reflection and transmission handled implicitly by their inclusion.

## 3. Results

Both the Tarigan and Xu/You simulations were reconstructed here, with comparisons and modifications, as noted, undertaken to reconcile any differences and to create a single, unified model. In addition, the reflection and transmission coefficients used by both methods were replaced by the full polarisation-variable formulations in amplitude rather than intensity. Lastly, a fast Fourier transform [22] and spectroscopic dechirping algorithm [18] were implemented to aid analysis. “Chirping” here refers to a monotonic increase in frequency with spatial position or angle, and is explained in greater detail later in this work.

The most striking result from the simulations is the difference between the fringe patterns seen for $\rho \;=\;\frac{r}{R}n_{1}\;<\;1$ and $\rho \;=\;\frac{r}{R}n_{1}\;>\;1$ capillaries. These refer to the cases where ray 5 does and does not contribute to the interference pattern seen, respectively. To distinguish these circumstances from each other, these situations will henceforth be known as low ρ and high ρ.

### 3.1. Intensity Patterns

**Figure 4:**
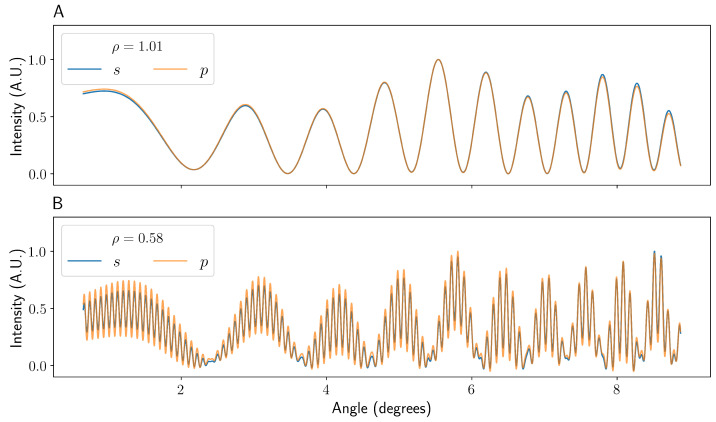
A graph showing the simulated intensity patterns seen for a capillary higher (**A**) and lower (**B**) than the ρ = 1 limit for both *s*- and *p*-polarised incident light. The data were taken 9 cm horizontally and 0.75 cm vertically from the capillary, with a simulated camera width of 1.3 cm. The angle is given in degrees from β = π. The data have been normalised between 0 and 1 to aid comparison.

**Figure 5:**
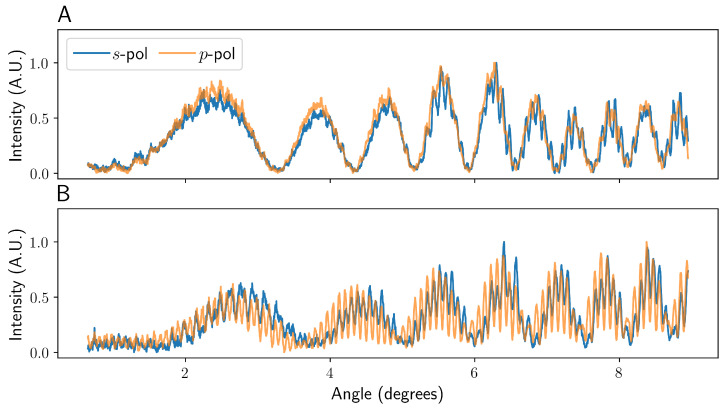
A figure showing the experimental intensity patterns seen for a capillary both higher (**A**) and lower (**B**) than the ρ = 1 limit. The data were taken at ∼9 cm horizontally and ∼0.75 cm vertically from the capillary, with a camera width of 1.3 cm. The data were longitudinally averaged to reduce high-frequency noise and produce a more representative fringe pattern [13]. The data have been normalised between 0 and 1 to aid comparison.

Figure 4 shows the simulated interference patterns for both *s*- and *p*-polarised incident light where ray 5 is not (A) and is (B) present across a range of viewing angles starting at β = π (directly backwards). Figure 5 shows line plots of experimentally obtained images with the same parameters as Figure 4, taken with the apparatus as described in the experimental methods.

As can be seen in Figure 4A and Figure 5A, the fringes formed at the camera in the case of high ρ resemble a two-beam interference pattern with a very low frequency envelope component. Specifically, the pattern is dominated by the interference between beam 1 and beam 4, with the modulations caused by the other terms. It can be seen in Figure 4A and Figure 5A that, both experimentally and in simulation, the imaged pattern is essentially polarisation-independent.

For low ρ values, the fringe pattern looks significantly different. As shown in Figure 4B and Figure 5B, the interference pattern where ρ is reduced from 1.01 to 0.58, but keeping otherwise identical parameters, shows a markedly higher frequency fringe pattern. This is due to the more rapid variation in the path length of beam 5 as a function of the incident angle. This high-frequency component stays in phase for both *s*- and *p*-polarised incident light, though the relative intensity is slightly lower for *s*-polarised light in the model presented here. The existence and variation with polarisation of these high-frequency fringes agree well with the model of Bornhop et al. [11]. The r/R and $n_{1}$ used in their simulation results are unclear, but are unlikely to result in ρ > 1. However, in a general experiment, the polarisation state used will have a negligible impact on the fringes observed, and so they make no difference to the final analysis or the quality of data. The low ρ fringe pattern looks more like a quintessential three-beam interference pattern. In this case, the fringes are dominated by the interference between beams 1, 4, and 5 with modulations by the other components. It should be noted that the slight relative shifts in the low-frequency oscillations shown in Figure 5, compared to the simulations in Figure 4, are most likely due to slight misalignment or manufacturing tolerances in the capillaries used.

### 3.2. Fourier Transforms

**Figure 6:**
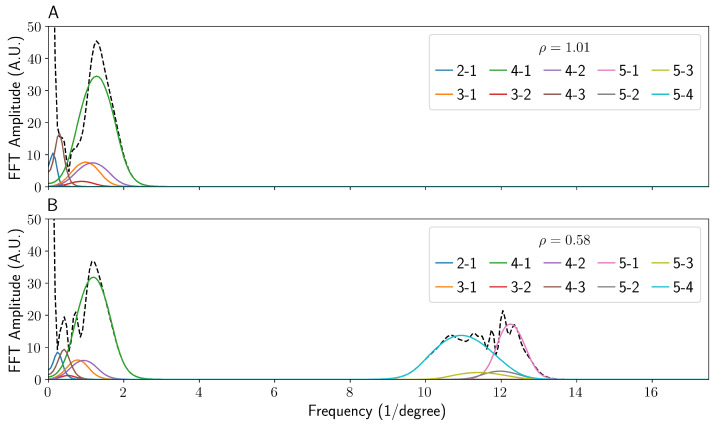
A graph comparing the Fourier transforms of both the high (**A**) and low (**B**) ρ values. The data were taken at 10 cm horizontally and 1 cm vertically from the capillary with a fixed camera width of 1.3 cm. The black dashed lines show the overall Fourier transforms of the patterns shown in Figure 4, with each pairwise interference term transformed and shown separately. For example, the interference between rays 1 and 2 is shown in blue and labelled 2-1. The data were windowed and zero-padded to reduce ringing and other artefacts after transformation.

The fast Fourier transforms for the simulated data of the two cases with *s*-polarised incident light are shown in Figure 6. The data for *p*-polarisation has been omitted as it is identical. The Fourier transforms for the experimental data can be found in the Supplementary Materials, and show excellent agreement. In the case typically seen in the literature, i.e., ρ > 1 (Figure 6A), the fringe pattern can be seen to be effectively a single frequency, dominated by the interference between ray 1 and 4. As ray 5 does not exist in this regime, any terms involving it are not present. At this particular choice of detector distance and vertical offset, many of the pairs of beams have a very similar frequency, leading to overlap. Due to this, the phase of this single peak should encode the information of how the refractive index of the liquid $n_{2}$ changes during the course of an experiment if monitored.

On the other hand, the Fourier transform of the low ρ case shown in Figure 6B shows an additional four high-spatial-frequency peaks, compared to the case of ρ > 1. Care must be taken if this set of fringes for phase analysis is used, due to the close proximity and comparable amplitude of a component that does (beam 5 with beam 4) and does not (beam 5 with beam 1) depend on the refractive index of the fluid in the capillary.

As can be seen in Figure 4 and Figure 5, the spatial frequency of the pattern projected onto the camera is not constant. As already briefly mentioned, this increase in spatial frequency with viewing angle is known as “chirping”, and is here caused by both the decreasing density of reflected rays as a function of incident angle as well as the projection of the angular pattern onto Euclidean space. For a given pair of rays, denoted by *i* and *j*, interfering in BSI, the increase or decrease in spatial frequency $\nu_{i}$ over viewing angle is linear, such that:

(6) $$\nu_{ij}\left(\beta \right)=\nu_{ij,0}+\alpha_{ij}\beta ,$$

where $\alpha_{ij}$ is the chirp rate and $\nu_{ij,0}$ the spatial frequency of the interference between the *i*th and *j*th ray at β = 0.

**Figure 7:**
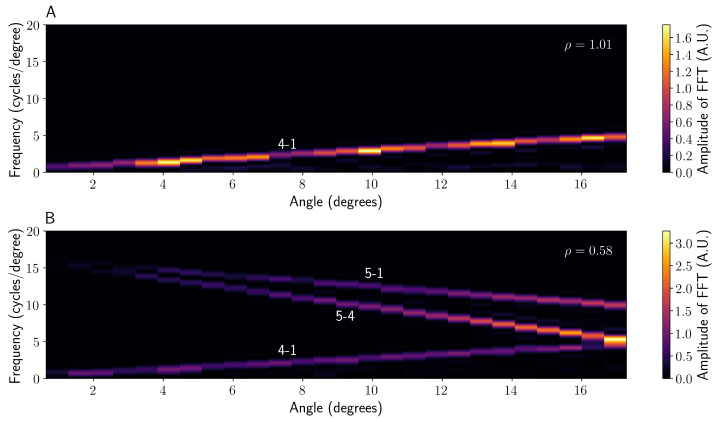
A spectrogram showing the rolling Fourier transform of the unchirped fringe pattern for both ρ > 1 (**A**) and ρ < 1 (**B**). Each distinct chirp rate $\alpha_{ij}$ is labelled with the corresponding beams that constitute it, e.g., 4-1 is the interference between rays 4 and 1.

This spatial frequency change over angle causes the peaks to become wider in Fourier space and, therefore, overlap, potentially leading to inaccurate phase monitoring. This can be corrected, for [18], through dechirping, assuming that a wide enough fringe section is analysed, by implementing a rolling Fourier transform or spectrogram as a function of viewing angle to determine $\alpha_{ij}$, as shown in Figure 7.

**Figure 8:**
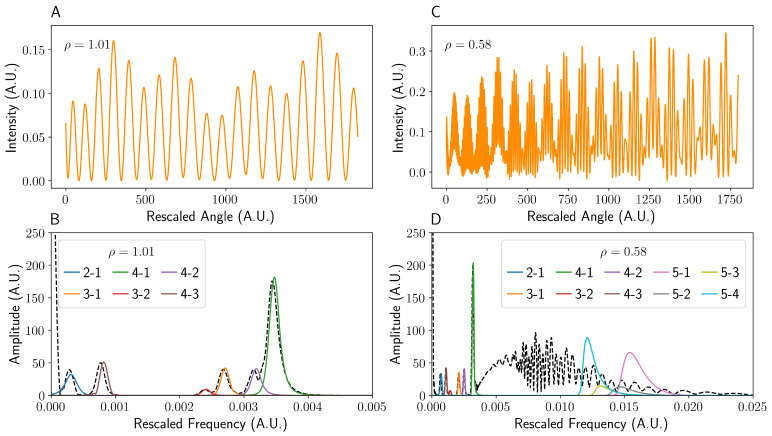
A series of graphs showing how dechirping of the interference pattern affects the Fourier domain. As can be seen in (**A**,**B**), the dechirping of the high ρ fringe pattern (**A**) leads to equally spaced fringes as well a Fourier transform that is the convolution of the pairwise interference terms (**B**). On the other hand, the dechirping of the low ρ intensity pattern (**C**) does not aid in the reconstruction of the beam 5 terms (**D**) due to its differing chirp frequency (see Figure 7). The black dashed line in (**B**,**D**) denotes the Fourier transform of the overall dechirped fringe patterns (**A**,**C**). For brevity, interference between beams *i* and *j* is denoted i−j. The data were windowed and zero-padded to reduce ringing and other artefacts after transformation, before finally dechirping.

When imaging large angular ranges (i.e., the camera is close to the capillary), it is essential to apply this chirping correction to obtain usable data. However, the magnitude and sign of $\alpha_{ij}$ varies for different beam components due to the unique angular conversion for each ray (see Supplementary Materials). In general, the lower spatial frequency interference components (i.e., 4-1 in Figure 6) increase in frequency with angle, whereas the higher spatial frequency components (such as 5-1 in Figure 6) tend to decrease, as can be seen in Figure 7. For a ρ > 1 system, implementing the dechirping causes the peaks in Fourier space for each pair of rays to become spatially resolved, yielding data identical to that given by Jørgensen et al. [18], as shown in Figure 8. Each interference term should, in fact, have a unique $\alpha_{ij}$, but in the case of ρ > 1, these chirp rates are sufficiently similar that they can be approximated as equal. Implementing the same dechirping for the ρ < 1 system, however, does not cause the interference fringes containing a component from ray 5 to become resolved. In fact, it causes the peaks to become less well-defined as the frequency of ray 5 decreases with increasing angle, contrary to rays 1–4 (see Figure 8). It is theoretically possible to dechirp multiple components of different $\alpha_{ij}$ values by using the product of the chirp rates $\prod_{ij}\alpha_{ij}$; however, this is only possible if they have the same sign (e.g., 5-1 and 5-4 in Figure 7B). Therefore, the value of ρ, combined with the frequencies of interest, should dictate the dechirping frequency $\alpha_{ij}$ used.

## 4. Discussion

### 4.1. Optimising BSI: Low ρ

To obtain the best Fourier transform in the region of low ρ using the setup defined in Section 2.1, the largest angular width possible should be interrogated to allow for more cycles and, therefore, greater frequency recognition. In addition, the chirping seen will become more pronounced, allowing better determination of the frequency shift that is needed to deconvolute it. As the chirp will consist of three $\alpha_{ij}$ components, extreme care must be taken to choose the correct one for the component chosen to monitor. In general, the detector used will have a fixed width. Therefore, to distinguish the Fourier components by interrogating the greatest angular range, it is advisable to place the detector as close as possible to the capillary. Care must, however, be taken to ensure the Nyquist criterion is fulfilled for the component of interest.

Ray 5 has a distinctly higher frequency, and does not traverse the sample ($n_{2}$), so it could be used to monitor environmental effects on the experiment. In Figure 6B, this would be component 5-1, for example. This interference component is distinct from the other high-amplitude peaks, and so by monitoring the phase of it, it may be possible to determine the refractive index change of the capillary itself, typically dominated by temperature fluctuations. This would be incredibly useful, as noise and drift in BSI measurements is often attributed to temperature instabilities [3,4,23]. As $dn_{1}/dT$ (where *T* is the temperature) is on the order of $10^{-6}$$\;\mathrm{RIU}\;K^{-1}$ [24], Equation (4) can be used to determine that ray 5 will shift by ∼$1.6\;\times \;10^{-2}$ path lengths per Kelvin, or equivalently, 0.1 radians per Kelvin with an inner and outer radius of r = 0.4 and R = 0.8. This number will scale with the size of the capillary used; therefore, incentivising the use of a larger capillary to allow a determination of the temperature change to a greater precision. To obtain a meaningful phase change for temperature changes on the order that Peltier devices are stable to, the capillary dimensions would need to be a factor of 100–1000× greater, which is impractical. The alternative is to construct the capillary from a material with a greater dn/dT value, ideally on the order of water itself ($dn/dT∼10^{-4}$$\;\mathrm{RIU}\;K^{-1}$), which could be accomplished by using a number of commercially available optical plastics [25].

### 4.2. Optimising BSI: High ρ

In the case of high ρ, the component of most interest is 4-1, which is by far the most dominant in the Fourier domain. Therefore, to determine the shift of the fringes (i.e., phase) with greater resolution, the detector can be placed far from the capillary as long as enough periods for the Fourier transform are present. By doing so, the phase of the interference pattern can be determined with a greater precision, as the physical shift in a fringe for a given refractive index change will be larger.

In general, having a system with high ρ is preferable due to it being more robust, less complicated to dechirp, and because it has a greater sensitivity due to a longer path length through the fluid. However, this is not to say that the case of low ρ is without merit or use, as has been discussed.

## 5. Conclusions

A systematic review and comparison of the available models surrounding ray tracing simulations of BSI has been presented. It was found that the importance of ray 5 in Figure 2, propagating within the capillary walls but not interacting with the sample, to the analysis of BSI fringe patterns had not been elucidated in prior literature. Nonetheless, conclusions drawn from these works remain mostly valid in the limits stated within this work. The ray tracing models were found to have discrepancies, but were contrasted, unified, and extended to include explicit polarisation dependence. Excellent agreement with experimental data is found, but the value of $\rho \;=\;n_{1}r/R$ must be selected with care and the proper analysis and dechirping are used in each case. The inclusion of ray 5 could allow for a concurrent temperature measurement of the capillary, though this may be reliant on different geometries or materials than those typically in use. Overall, it is suggested that the most common case of ρ > 1 continues to be used with the detector placed far away, unless a suitable capillary with a high dn/dT value is found. This work highlights the importance of a thorough understanding of the multiple interference components to facilitate unambiguous interpretation of BSI data.

## Data Availability

The data presented in this study are available in the Article and Supplementary Materials.

## Supplementary Materials

The following supporting information can be downloaded at: https://www.mdpi.com/article/10.3390/s22062157/s1, Figure S1: Fourier transforms of experimental fringe patterns. Equations (S1)–(S35): equations for full model including transmittance/reflectance, angles, amplitude coefficients, and path lengths for rays 1–7.
