# Supplementary material for: Shedding Light on Capillary-Based Backscattering Interferometry
Source: Sensors (Basel). 2022 Mar 10;22(6):2157. doi: 10.3390/s22062157 (PMC8949530; doi:10.3390/s22062157)
Supplement: Supplementary file 1 [file sensors-22-02157-s001.zip › sensors-1614501-supplementary.pdf]

Supplementary Information - Shedding Light on Capillary-Based Backscattering Interferometry

Niall M. C. Mulkerns,[†,‡] William H. Hoffmann,[†,‡,¶] Ian D. Lindsay,[†,‡] and Henkjan Gersen[*,†,‡]

†*H. H. Wills Physics Laboratory, University of Bristol, Bristol, BS8 1TL, United Kingdom*

‡*Bristol Centre for Functional Nanomaterials, University of Bristol, Bristol, BS8 1TL, United Kingdom*

¶*School of Chemistry, University of Bristol, Bristol, BS8 1TS, United Kingdom*

E-mail: h.gersen@bristol.ac.uk

Experimental Fourier Transforms

Fourier transforms of the data given in Fig. 5 in the main text are shown in Fig. S1. When compared to the simulated data shown in Fig. 6, it is possible to see that both the frequency and number of the peaks are in excellent agreement, demonstrating the ability of the model presented here to describe the system.

Full Model

Unless stated otherwise, the variables used here are the same as defined in the main body of text. The transmittance t_r and reflectance r_e of a given ray in terms of amplitude are

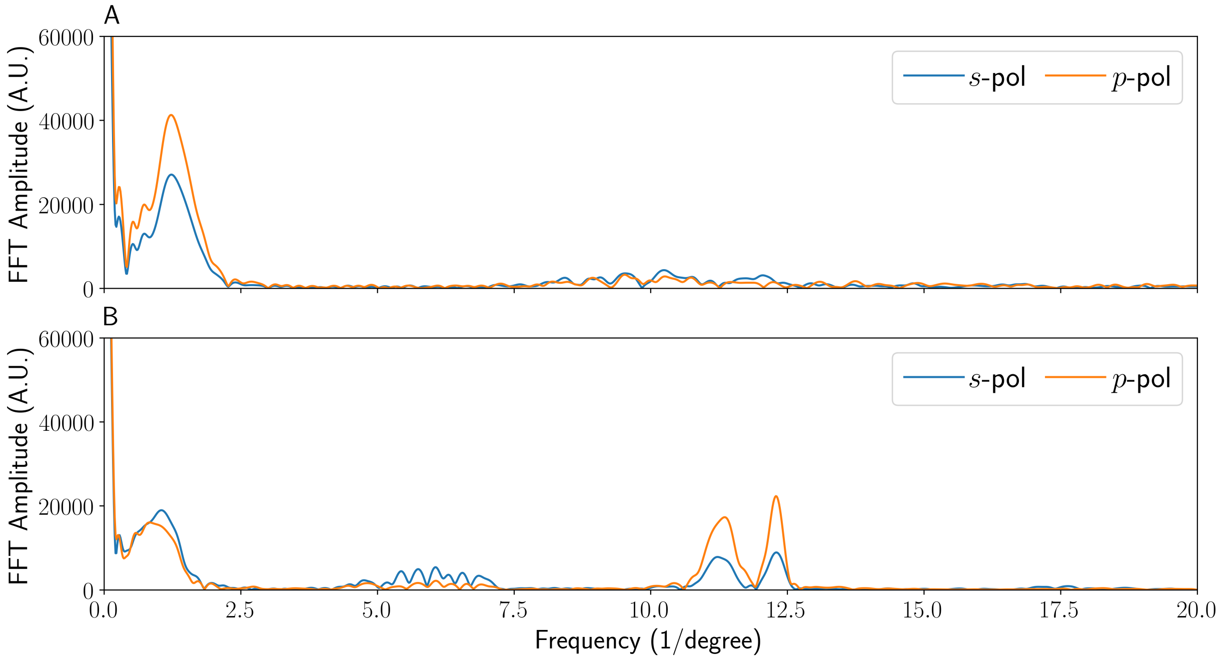

Figure S1: A graph comparing the Fourier transforms of both the high (A) and low (B) ρ data obtained experimentally for both s- and p-polarised incident light. The data, shown in Fig. 5 in the main text, were taken at $\sim 9\,\text{cm}$ horizontally and $\sim 0.75\,\text{cm}$ vertically from the capillary, with a camera width of 1.3 cm. The data were windowed and zero-padded to reduce ringing and other artefacts after transformation.

described by

$$t_r = \sin(\psi)\left[\frac{2\cos(A)\sin(B)}{\sin(A+B)}\right] + \cos(\psi)\left[\frac{2\cos(A)\sin(B)}{\sin(A+B)\cos(A-B)}\right] \tag{S1}$$

$$r_e = -\sin(\psi)\left[\frac{\sin(A-B)}{\sin(A+B)}\right] + \cos(\psi)\left[\frac{\tan(A-B)}{\tan(A+B}\right], \tag{S2}$$

where A and B are the incident and refracted angles respectively, and ψ is the angle of the polariser. The refracted angles can be calculated using Snell's law.

The scattering factors in amplitude are as follows:

$$s_1 = r_e(\phi_1, n_0, n_1) \tag{S3}$$

$$s_2 = t_r(\phi_2, n_0, n_1) r_e(\delta_2, n_1, n_2) t_r(\theta_2, n_1, n_0) \tag{S4}$$

$$s_3 = t_r(\phi_3, n_0, n_1) t_r(\delta_3, n_1, n_2) t_r(\epsilon_3, n_2, n_1) r_e(\epsilon_3, n_2, n_1) t_r(\theta_3, n_1, n_0) \tag{S5}$$

$$\begin{aligned} s_4 = {} & t_r(\phi_4, n_0, n_1) t_r(\delta_4, n_1, n_2) t_r(\epsilon_4, n_2, n_1) r_e(\delta_4, n_1, n_0) \\ & t_r(\theta_4, n_1, n_2) t_r(\epsilon_4, n_2, n_1) t_r(\theta_4, n_1, n_0) \end{aligned} \tag{S6}$$

$$s_5 = t_r(\phi_5, n_0, n_1) r_e(\theta_5, n_1, n_0) t_r(\theta_5, n_1, n_0) \tag{S7}$$

$$s_6 = t_r(\phi_6, n_0, n_1) t_r(\theta_6, n_1, n_0) \tag{S8}$$

$$s_7 = t_r(\phi_7, n_0, n_1) t_r(\delta_7, n_1, n_2) t_r(\epsilon_7, n_2, n_1) t_r(\theta_7, n_1, n_0) \tag{S9}$$

where

$$\theta_i = \arcsin\left(\frac{\sin(\phi_i)}{n_1}\right) \tag{S10}$$

$$\delta_i = \arcsin\left(\frac{R\sin(\phi_i)}{rn_1}\right) \tag{S11}$$

$$\epsilon_i = \arcsin\left(\frac{R\sin(\phi_i)}{rn_2}\right). \tag{S12}$$

The scattering factor in power is determined simply by squaring the appropriate factor in amplitude (*i.e.* $S_i = s_i^2$). Note that the f_i correction factor should be applied after this.

The angular transformations are found to be

$$\beta_1 = 2\phi_1 - \pi \tag{S13}$$

$$\beta_2 = 2(\phi_2 - \theta_2 + \delta_2) - \pi \tag{S14}$$

$$\beta_3 = 2(\phi_3 - \theta_3 + \delta_3 - 2\epsilon_3) + \pi \tag{S15}$$

$$\beta_4 = 2(\phi_4 - 2\theta_4 + 2\delta_4 - 2\epsilon_4) + \pi \tag{S16}$$

$$\beta_5 = 2(\phi_5 - 2\theta_5) + \pi \tag{S17}$$

$$\beta_6 = 2(\phi_6 - \theta_6) \tag{S18}$$

$$\beta_7 = 2(\phi_7 - \theta_7 + \delta_7 - \epsilon_7) \tag{S19}$$

with their respective limits set by

$$0 \leq \quad \beta_1 \quad \leq \pi/2 \tag{S20}$$

$$0 \leq \quad \beta_2 \quad \leq \arcsin(rn_1/R) \tag{S21}$$

$$0 \leq \quad \beta_3 \quad \leq \arcsin(rn_2/R) \tag{S22}$$

$$0 \leq \quad \beta_4 \quad \leq \arcsin(rn_2/R) \tag{S23}$$

$$\arcsin(rn_1/R) \leq \quad \beta_5 \quad \leq \pi/2 \tag{S24}$$

$$\arcsin(rn_1/R) \leq \quad \beta_6 \quad \leq \pi/2 \tag{S25}$$

$$0 \leq \quad \beta_4 \quad \leq \arcsin(rn_2/R) \tag{S26}$$

The path lengths are defined as follows. For simplicity, it is assumed that the refractive

index of the medium surrounding the capillary is unity.

$$L_1 = 2R[1 - \cos(\phi_1)] \tag{S27}$$

$$L_2 = 2R[1 - \cos(\phi_2)] + 2n_1 R\cos(\theta_2) - 2n_1 r\cos(\delta_2) \tag{S28}$$

$$L_3 = 2R[1 - \cos(\phi_3)] + 2n_1 R\cos(\theta_3) - 2n_1 r\cos(\delta_3) + 4n_2 r\cos(\epsilon_3) \tag{S29}$$

$$L_4 = 2R[1 - \cos(\phi_4)] + 4n_1 R\cos(\theta_4) - 4n_1 r\cos(\delta_4) + 4n_2 r\cos(\epsilon_4) \tag{S30}$$

$$L_5 = 2R[1 - \cos(\phi_5)] + 4n_1 R\cos(\theta_5) \tag{S31}$$

$$L_6 = 2R[1 - \cos(\phi_6)] + 2n_1 R\cos(\theta_6) \tag{S32}$$

$$L_7 = 2R[1 - \cos(\phi_7)] + 2n_1 R\cos(\theta_7) - 2n_1 r\cos(\delta_7) + 2n_2 r\cos(\epsilon_7). \tag{S33}$$

Conversions between the spatial position and the angular position of the detector were calculated using

$$\beta_{min} = \arctan\left(\frac{h}{d}\right), \tag{S34}$$

and

$$\Delta\beta = \arctan\left(\frac{c}{d}\right), \tag{S35}$$

where h is the vertical distance between the centre of the capillary and the centre of the detector, c is the physical dimension of the detector used for detection, and d is the horizontal distance from the capillary to the detector. From known or assumed h, d, and c values, the minimum viewing angle (β_{min}) and the maximum viewing angle ($\beta_{min} + \Delta\beta$) can be determined and used in calculations.
